# Validity of a four-item questionnaire in French assessing attachment to meat

**DOI:** 10.3389/fmed.2024.1383825

**Published:** 2024-10-04

**Authors:** Paul Sebo, Benoit Tudrej, Augustin Bernard, Bruno Delaunay, Alexandra Dupuy, Claire Malavergne, Hubert Maisonneuve

**Affiliations:** ^1^University Institute for Primary Care (IuMFE), University of Geneva, Geneva, Switzerland; ^2^University College of General Medicine, University Claude Bernard Lyon 1, Lyon, France

**Keywords:** attachment to meat, french, MAQ, meat attachment questionnaire, primary care, validation

## Abstract

**Background:**

The 16-item Meat Attachment Questionnaire (MAQ-16) assesses emotional and cognitive attachment to meat across four dimensions: hedonism, affinity, entitlement, and dependence. Recently validated in French, we aimed to develop and validate a shorter, four-item version (MAQ^f^-4) to reduce participant burden.

**Methods:**

In this 2023 observational study in the Rhône-Alpes region, 919 primary care patients were invited to complete the French MAQ-16 (MAQ^f^-16). Classical test theory guided the development of the MAQ^f^-4, and Spearman’s correlation coefficients assessed its correlation with the MAQ^f^-16 (dimension and overall scores). We also evaluated the diagnostic performance of the MAQ^f^-4 for identifying patients with high meat attachment (MAQ^f^-16 score > 60).

**Results:**

A total of 822 patients participated (65.3% women; median age = 52; participation rate = 89.5%). The MAQ^f^-4 showed strong correlations with the MAQ^f^-16 (rho = 0.83 for hedonism, 0.77 for affinity, 0.70 for entitlement, 0.79 for dependence, and 0.86 for the overall score, all *p*-values <0.001). A score < 15 on the MAQ^f^-4 (sensitivity = 91%, NPV = 96%) effectively excluded patients with low meat attachment, while a score ≥ 17 (specificity = 96%, PPV = 84%) accurately identified those with high attachment.

**Conclusion:**

The MAQ^f^-4 demonstrated strong correlation with the MAQ^f^-16 and accurately identified high attachment to meat. It may serve as a useful tool in research and clinical settings, though further validation is required before broad implementation in French primary care.

## Introduction

In recent years, increasing attention has been given to the environmental, ethical, and health implications of meat consumption. As dietary habits evolve, particularly with the promotion of plant-based diets, understanding the psychological and cultural attachment individuals have to meat is crucial. Assessing this attachment can inform both public health strategies and individual interventions aimed at reducing meat consumption.

The Meat Attachment Questionnaire (MAQ-16) is a widely used 16-item psychometric tool that explores four dimensions of meat attachment: hedonism, affinity, entitlement, and dependence (1). It was designed to assess individuals’ emotional and cognitive connections to meat consumption and aligns with behavior change theories in social psychology, particularly within the Behavior Change Technique Taxonomy (2). As research on the psychological factors influencing dietary preferences expands, the MAQ-16 has become an essential tool for studying meat-eating behavior (3–7).

Originally developed and validated by Graça in English and Portuguese (1), the MAQ-16 has also been used in other languages, such as Dutch and Finnish (8), although, to our knowledge, no validation studies have been conducted in these contexts. Recently, our research team translated the MAQ-16 into French and demonstrated its reliability and validity among French-speaking primary care patients (9). However, to streamline data collection and optimize questionnaire administration, particularly in settings like primary care consultations, we aimed to develop a shorter version of the MAQ-16.

The goal of this study was to develop and validate a concise, four-item version of the MAQ-16 in French (one item per dimension), ensuring it retained accuracy in measuring attachment to meat while minimizing the burden on participants. This study is part of a broader project examining meat consumption patterns in primary care patients, with the aim of developing targeted interventions to reduce meat consumption.

The main contribution of this study lies in the development and validation of a concise, four-item version of the Meat Attachment Questionnaire, specifically adapted for French-speaking populations. This shorter version retains the psychometric properties of the original 16-item scale while offering a more practical tool for use in both research and clinical setting. Our study not only validates this four-item version but also establishes its potential for use in large-scale studies, particularly in contexts where time constraints limit the feasibility of longer instruments. More broadly, our research contributes to the growing body of literature on dietary behavior and highlights the importance of tailored interventions to promote healthier dietary habits, particularly given the well-documented health (10–13) and environmental (14–16) consequences associated with excessive meat consumption.

## Methods

### Study setting

**Figure 1:**
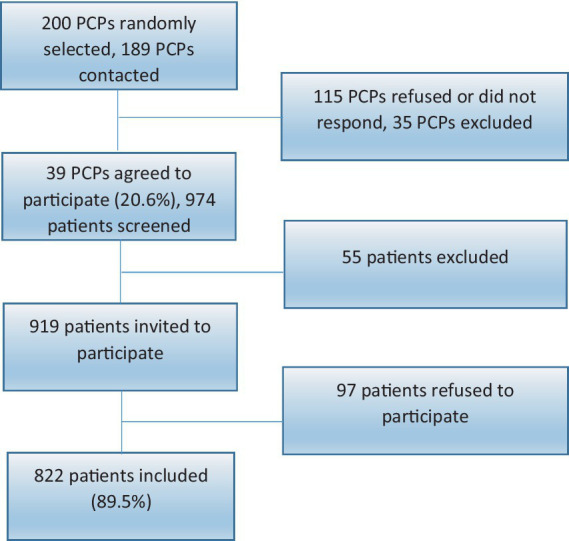
Flowchart of the study.

This observational study was conducted in 2023 among French primary care patients. Figure 1 illustrates the recruitment process. Patients were recruited in two stages. First, we randomly selected 39 physicians from a professional register of primary care physicians (PCPs) in the Rhône-Alpes region. We then recruited a sample of 919 consecutive non-urgent patients from the waiting rooms of these 39 physicians. These patients were asked to complete the French version of the MAQ-16 while in the waiting room. All participants provided informed consent before taking part in the study. Ethical approval was obtained from the Research Ethics Committee of the University Claude Bernard Lyon (Project-ID=IRB2023-01-03-01).

### Meat attachment questionnaire (MAQ-16)

The 16-item Meat Attachment Questionnaire (MAQ-16) measures four dimensions of individuals’ relationship with meat consumption: hedonism, affinity, entitlement, and dependence (1). The questionnaire is rated on a 5-point Likert scale, with total scores ranging from 16 to 80. Items #4, #6, #9, #13, and #14 are reverse-coded. A higher total score indicates a stronger attachment to meat. For the French validation of the MAQ-16, we developed a version with 17 items (MAQ^f^-17), splitting item #15 into two separate questions: item #15a (“eating meat is a natural practice”) and item #15b (“eating meat is an indisputable practice”). This version was recently validated by our research team (9).

### Development and validation of the four-item MAQ in French (MAQ^f^-4)

To simplify the questionnaire for use in primary care, we developed a four-item version of the MAQ in French (MAQ^f^-4). We based this on the 16-item version (with item #15a), as the MAQ^f^-16 and MAQ^f^-17 showed a perfect correlation (Spearman’s rank correlation coefficient = 1.00, *p*-value <0.001), indicating that item #15b added no additional value.

Using Stata’s ‘validscale’ command (17), which applies classical test theory (CTT) (18), we evaluated the psychometric properties of the questionnaire. Initially, we selected two items per dimension based on their highest Cronbach’s alpha (indicating internal consistency) and Loevinger’s H coefficient (indicating scalability) (19, 20). Minimum acceptable values for Cronbach’s alpha and Loevinger’s H coefficient were 0.70 and 0.30, respectively (19, 20). After internal discussions, we selected one item per dimension based on clarity and its ability to best represent the dimension. The final MAQ^f^-4 consists of items #5, #12, #14, and #15a.

### Statistical analyses

We assessed the validity of the MAQ^f^-4 by calculating Spearman’s rank correlation coefficients between the MAQ^f^-4 and the validated MAQ^f^-16, both for individual dimensions and overall scores. Correlations of 0.10 were considered ‘small’, 0.30 ‘moderate’, and 0.50 ‘large’ (21). Internal consistency was evaluated using Cronbach’s alpha, and scalability was assessed with Loevinger’s H coefficient. In this preliminary study, we did not examine the test–retest reliability of the instrument.

To evaluate the diagnostic performance of the MAQ^f^-4 in identifying patients with high attachment to meat, we dichotomized patients into two groups: ‘high attachment to meat’ and ‘medium/low attachment to meat’, using the 75^th^ percentile of the MAQ^f^-16 score as the cutoff, as recommended by other studies (22). The diagnostic performance of the MAQ^f^-4 was assessed using Stata’s ‘roctab’ command to compute sensitivity, specificity, the area under the receiver operating characteristic (ROC) curve, and positive and negative predictive values (PPVs and NPVs) for different cutoffs values.

We conducted subgroup analyses by gender (men and women) and age group (patients under and over 50 years old) and used logistic regression, adjusted for intra-cluster correlation within practices, to examine significant differences in the proportion of patients with high attachment to meat. All analyses were performed using STATA 15.1 (College Station, TX).

## Results

A total of 822 patients (65.3% women; median age = 52, interquartile range = 31, age range = 20–93) agreed to participate in the study, yielding a participation rate of 89.5%.

**Figure 2:**
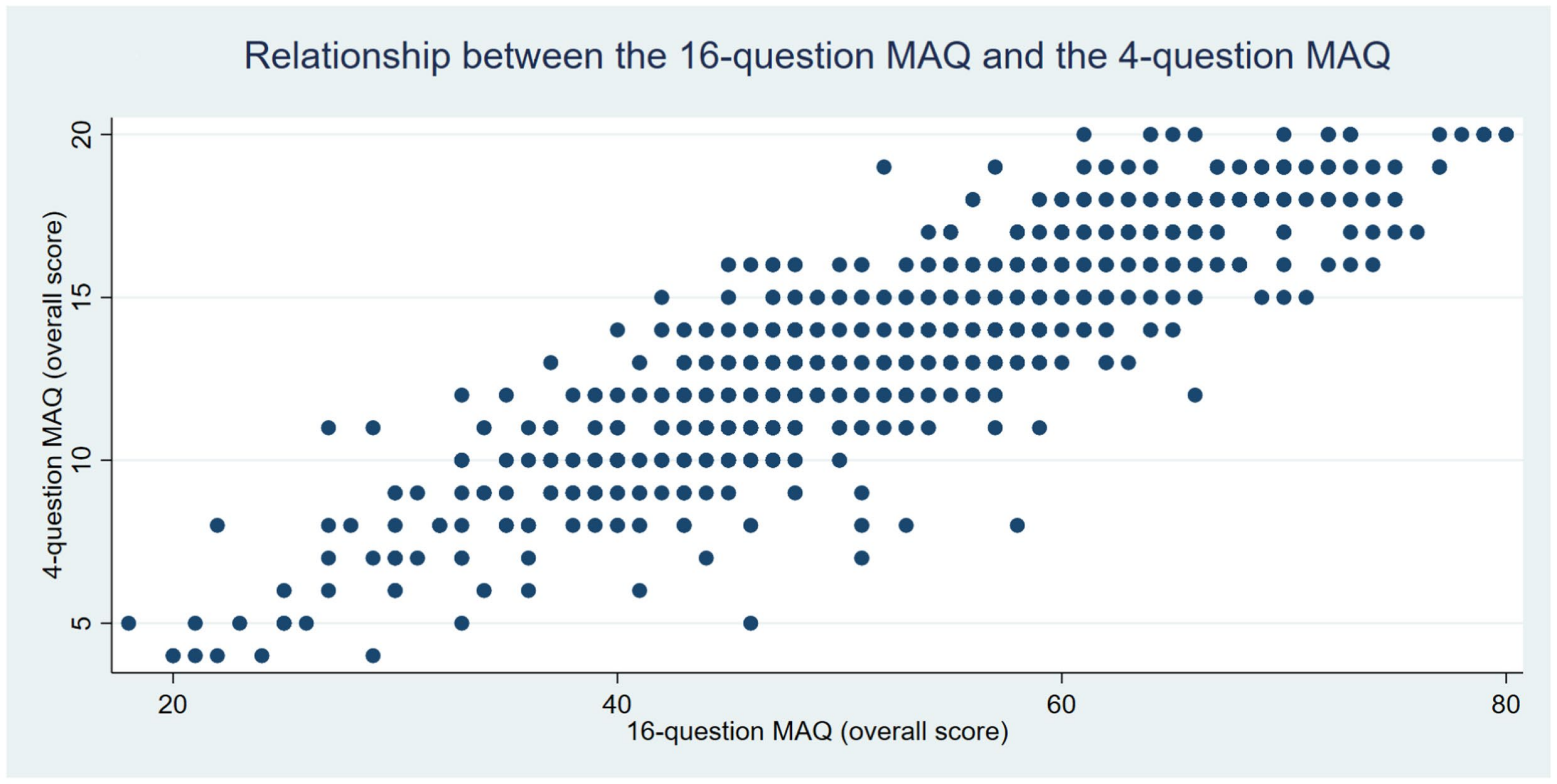
Scatterplot showing the overall score for the four-item French ‘Meat Attachment Questionnaire’ (MAQ^f^-4) versus the 16-item ‘Meat Attachment Questionnaire’ (MAQ^f^-16).

Correlations between the MAQ^f^-4 and MAQ^f^-16 were strong across all four dimensions and for the overall score (rho = 0.83 for *hedonism*, 0.77 for *affinity*, 0.70 for *entitlement*, 0.79 for *dependence*, and 0.86 for the overall score, all *p* values <0.001). The scatterplot for the overall score is presented in Figure 2. These correlations were similar between men and women (overall score: rho = 0.85 and 0.86, respectively) and between patients aged below and above 50 years (overall score: rho = 0.87 and 0.86, respectively). The MAQ^f^-4 demonstrated acceptable internal consistency (Cronbach’s alpha = 0.78) and scalability (Loevinger’s H coefficient = 0.34).

Of the participants, 200 patients (24.8%) had an overall MAQ^f^-16 score greater than 60, indicating high attachment to meat. Men were significantly more likely to have high attachment (99/279, 35.5%) compared to women (101/528, 19.1%, *p*-value <0.001). No significant association was observed with age (under 50 years: 99/379, 26.1% vs. 100/426, 23.5%, *p*-value = 0.30).

Table 1 displays the diagnostic performance of the MAQ^f^-4 across various thresholds. The highest overall performance was observed at a cutoff of 16 (ROC area = 0.85, sensitivity = 81.0%, specificity = 88.5%) and 15 (ROC area = 0.84, sensitivity = 91.0%, specificity = 77.3%). Performance was similar across gender and age groups (ROC area = 0.83–0.85). PPVs were highest for cutoffs of 18 (91.6%) and 17 (84.1%), while NPVs were highest for cutoffs of 15 (96.3%) and 16 (93.4%), with consistency across gender and age groups (Table 1).

**Table 1:** Diagnostic performance of the four-item French ‘Meat Attachment Questionnaire’ (MAQ^f^-4) in identifying patients with high attachment to meat (overall score > 60 on the 16-item French ‘Meat Attachment Questionnaire’).

Overall score for the MAQ^f^-4	Number of patients with a high attachment to meat according to the overall score for the MAQ^f^-4, n/N (%)	Sensitivity (95%CI)	Specificity (95%CI)	ROC area (95%CI)	Positive predictive value (95%CI)	Negative predictive value (95%CI)
**Overall sample**						
Score ≥ 15	324 / 818 (39.6)	91.0 (86.1–94.6)	77.3 (73–8-80.6)	0.84 (0.82–0.87)	56.9 (51.2–62.4)	96.3 (94.2–97.8)
Score ≥ 16	235 / 818 (28.7)	81.0 (74.9–86.2)	88.5 (85.7–90.9)	0.85 (0.82–0.88)	69.8 (63.5–75.7)	93.4 (91.1–95.3)
Score ≥ 17	158 / 818 (19.3)	66.0 (59.0–72.5)	95.9 (94.0–97.3)	0.81 (0.78–0.84)	84.1 (77.4–89.4)	89.6 (86.9–91.8)
Score ≥ 18	96 / 818 (11.7)	43.5 (36.5–50.7)	98.7 (97.4–99.4)	0.71 (0.68–0.75)	91.6 (84.1–96.3)	84.2 (81.3–86.8)
**Men**						
Score ≥ 15	137 / 283 (48.4)	90.9 (83.4–95.8)	75.6 (68.6–81.6)	0.83 (0.79–0.88)	67.2 (58.5–75.0)	93.8 (88.5–97.1)
Score ≥ 16	108 / 283 (38.2)	81.8 (72.8–88.9)	86.1 (80.2–90.8)	0.84 (0.79–0.89)	76.4 (67.2–84.1)	89.6 (84.1–93.7)
Score ≥ 17	80 / 283 (28.3)	71.7 (61.8–80.3)	95.0 (90.7–97.7)	0.83 (0.79–0.88)	88.8 (79.7–94.7)	85.9 (80.3–90.4)
Score ≥ 18	49 / 283 (17.3)	45.5 (35.4–55.8)	97.8 (94.4–99.4)	0.72 (0.67–0.77)	91.8 (80.497.7)	76.5 (70.5–81.8)
**Women**						
Score ≥ 15	187 / 534 (35.0)	91.1 (83.8–95.8)	78.0 (73.8–81.8)	0.85 (0.81–0.88)	49.5 (42.1–56.9)	97.4 (95.1–98.8)
Score ≥ 16	127 / 534 (23.8)	80.2 (71.1–87.5)	89.5 (86.2–92.2)	0.85 (0.81–0.89)	64.3 (55.3–72.6)	95.0 (92.4–96.9)
Score ≥ 17	78 / 534 (14.6)	60.4 (50.2–70.0)	96.3 (94.0–97.8)	0.78 (0.73–0.83)	79.2 (68.5–87.6)	91.1 (88.1–93.6)
Score ≥ 18	47 / 534 (8.8)	41.6 (31.9–51.8)	99.1 (97.6–99.7)	0.70 (0.66–0.75)	91.3 (79.2–97.6)	87.8 (84.5–90.5)
**<50 years old**						
Score ≥ 15	181 / 379 (47.8)	96.0 (90.0–98.9)	69.3 (63.5–74.6)	0.83 (0.79–0.86)	52.5 (44.9–59.9)	98.0 (94.9–99.4)
Score ≥ 16	121 / 379 (31.9)	83.8 (75.1–90.5)	86.4 (81.9–0.90.2)	0.85 (0.81–0.89)	68.6 (59.5–76.7)	93.8 (90.1–96.4)
Score ≥ 17	84 / 379 (22.2)	69.7 (59.6–78.5)	94.6 (91.3–97.0)	0.82 (0.77–0.87)	82.1 (72.3–89.6)	89.8 (85.8–93.0)
Score ≥ 18	50 / 379 (13.2)	47.5 (37.3–57.8)	98.9 (96.9–99.8)	0.73 (0.68–0.78)	94.0 (83.5–98.7)	84.2 (79.8–88.0)
**≥50 years old**						
Score ≥ 15	141 / 436 (32.3)	86.0 (77.6–92.1)	84.4 (79.9–88.1)	0.85 (0.81–0.89)	62.8 (54.1–70.9)	95.2 (92.0–97.3)
Score ≥ 16	113 / 436 (25.9)	78.0 (68.6–85.7)	90.2 (86.4–93.2)	0.84 (0.80–0.89)	70.9 (61.5–79.2)	93.0 (89.6–95.6)
Score ≥ 17	74 / 436 (17.0)	63.0 (52.8–72.4)	96.9 (94.4–98.5)	0.80 (0.75–0.85)	86.3 (76.2–93.2)	89.5 (85.8–92.5)
Score ≥ 18	46 / 436 (10.6)	40.0 (30.3–50.3)	98.5 (96.5–99.5)	0.69 (0.64–0.74)	88.9 (75.9–96.3)	84.3 (80.2–87.8)

Results are presented for the overall sample and four subgroups (men, women, under 50, over 50) across four score thresholds.

## Discussion

### Main findings

In this study of French primary care patients, we found that the MAQ^f^-4 correlated strongly with the validated MAQ^f^-16 and accurately identified patients with high attachment to meat, both in the overall sample and in sub-samples (men/women, patients under/over 50). We also found that men were overrepresented among patients with high meat attachment, consistent with results reported by Graça et al. (1).

### Comparison with existing literature

The MAQ^f^-4 can effectively assess individuals’ emotional and cognitive connections to meat consumption, with strong correlation to the MAQ^f^-16 (rho = 0.86). The higher the score, the stronger the attachment to meat. A threshold of 15 yielded a sensitivity of 91% and a NPV of 96%, which accurately excluded patients with no meat attachment (score < 15). Among the 200 patients with a high meat attachment (overall score > 60 on the MAQ^f^-16), only 18 were false negatives. For patients with high attachment (score ≥ 17), the specificity was 96%, and the PPV was 84%. There were only 25 false positives among patients with medium/low attachment. As PPVs are influenced by the prevalence of meat attachment (set at 25% overall), PPVs would be expected to rise in populations with higher attachment, as observed in male patients (PPV = 89% using a threshold of 17).

The MAQ-16 has been widely used in various studies to explore meat attachment (1, 3–8, 23). Its ability to discriminate between different levels of attachment provides valuable insights into dietary behaviors. The development of the MAQ-4 in French (MAQ^f^-4) marks significant progress in dietary behavior research, particularly in primary care. By simplifying the original questionnaire, the MAQ^f^-4 offers a practical tool for evaluating individuals’ emotional and cognitive connections to meat consumption.

### International relevance

Although this study was conducted in a French population, the findings have broader implications for international research on dietary behaviors. Given global concerns over meat consumption’s impact on health (10–13) and the environment (14–16), the MAQ^f^-4 could be adapted and validated in other languages and cultural contexts. Future studies should focus on cross-cultural validation, particularly in regions with higher meat consumption. The MAQ^f^-4 could contribute to global efforts to promote more sustainable and health-conscious eating patterns.

### Clinical implications

From a clinical perspective, the MAQ^f^-4 is well-suited for use in primary care setting due to its brevity and ease of administration. It could facilitate discussions about dietary habits and help healthcare providers identify patients with strong meat attachment. A two-step approach, where the MAQ^f^-4 is used for initial screening followed by the MAQ^f^-16 for more detailed assessments, could provide deeper insights into the psychological drivers of meat consumption and help tailor interventions. This approach may enhance understanding of the health (10–13) and environmental impacts (14–16) of meat consumption.

### Methodological considerations and future research

In our study, we used Stata’s ‘validscale’ command based on classical test theory (CTT) to develop and validate the MAQ^f^-4. While ‘validscale’ effectively assessed key psychometric properties such as internal consistency and scalability, it did not cover other important psychometric indices. Future studies should incorporate these additional measures to provide a more comprehensive validation of the MAQ^f^-4. Moreover, test–retest reliability was not assessed in this study and should be prioritized in future research. However, the development of the four-item questionnaire was based on the MAQ^f^-16, which has already been validated by our research team. As such, we considered further psychometric analysis to be less critical at this stage.

## Limitations

Several limitations should be acknowledged. First, this study was conducted in a single region of France, limiting the generalizability of the findings to other French-speaking populations or cultural contexts. Second, while internal consistency was found to be acceptable (Cronbach’s alpha = 0.78), test–retest reliability was not evaluated. Third, the study did not explore how well the MAQ^f^-4 predicts actual meat consumption, warranting further research on this relationship. Finally, more studies on convergent and divergent validity are needed to better understand how the MAQ^f^-4 relates to related constructs, such as dietary patterns and psychological factors.

## Conclusion

The MAQ^f^-4 is a promising tool for measuring meat attachment, correlating well with the MAQ^f^-16 while minimizing participant burden. It has clear potential for use in both research and clinical settings to assess dietary behaviors, but further validation in other cultural contexts and studies examining its relationship with actual meat consumption are required. The MAQ^f^-4 represents an important step in advancing research on dietary behavior and promoting more sustainable and health-conscious eating patterns.

## Data Availability

The raw data supporting the conclusions of this article will be made available by the authors, without undue reservation.
